# Progress on the Prevention of Esophageal Stricture after Endoscopic Submucosal Dissection

**DOI:** 10.1155/2018/1696849

**Published:** 2018-03-04

**Authors:** Peina Shi, Xiaoyun Ding

**Affiliations:** ^1^Medical School, Ningbo University, Ningbo, Zhejiang, China; ^2^Gastroenterology Department, Ningbo First Hospital, Ningbo, Zhejiang, China

## Abstract

Endoscopic submucosal dissection (ESD) has been widely accepted as an effective, minimally invasive treatment for superficial esophageal cancers. However, esophageal stricture often occurs in patients with large mucosal defects after ESD. In this review, we discuss various approaches recently researched to prevent esophageal strictures after ESD. These approaches can be classified as pharmacological treatments, esophageal stent treatments, and tissue engineering approaches. Most of the preventive approaches still have their limitations and require further research. With the improvement of current therapies, ESD can be more widely utilized as a minimally invasive treatment with minimal complications.

## 1. Introduction

Currently, endoscopic submucosal dissection (ESD) has been widely accepted as an effective, minimally invasive treatment for superficial esophageal cancers, including esophageal squamous cell carcinoma and Barrett's adenocarcinoma [1–3]. Compared to endoscopic mucosal resection (EMR), ESD has substantially higher en bloc and curative resection rates, lower local recurrence rates, and more precise histopathologic assessment [4, 5]. However, esophageal stricture often occurs inevitably in patients who have large mucosal defects after ESD. Esophageal strictures cause nausea, vomiting, and varying degrees of dysphagia and may influence patients' vocalization, severely decreasing the patient's quality of life. Patients with esophageal strictures require multiple endoscopic balloon dilations (EBDs) or dilations with a bougie over a long period [6]. Multiple sessions of endoscopic dilation are painful and increase the risk of esophageal perforation [7, 8]. Therefore, various kinds of approaches to prevent esophageal strictures after ESD are necessary and useful.

Esophageal stricture after ESD can be structurally divided into two categories: (1) a simple stricture, meaning that the stricture is short, focal, and not angulated and has a diameter that will allow the endoscope to pass, and (2) a complex stricture, meaning that the stricture is long (>2 cm), irregular, and angulated or has a severely narrow diameter [9]. In clinical research, esophageal stricture after ESD often refers to the complex stricture, and the patient has a feeling of dysphagia or the stricture prevents the passage of a standard 9.2–10 mm diameter endoscope. Two main mechanisms can explain the esophageal stricture after ESD: (1) the loss of the esophageal epithelium, which means the loss of a barrier against saliva, gastric acid, microorganisms, and so on, and (2) inflammation, fibrosis, and scar formation in the process of wound healing [10]. The rate of stricture occurrence after near-circumference or whole-circumference ESD was reported to be 88–100% [2, 9, 11, 12]. Esophageal stents, extracellular matrix scaffolds, and cell-based therapy have been researched to address this problem. The severe inflammation is due to the stimulation of several physical and chemical factors, as well as to the after-effects of the heat damage caused by the use of a high-frequency wave snare [13]. This inflammation results in ulceration of the deep layer of the esophagus. Myofiber atrophy and fibrosis reactions gradually appear during the period of the wound recovery, which finally results in esophageal stricture [13, 14]. Steroids and some antifibrotic drugs have been reported to mitigate these types of reactions.

## 2. Pharmacological Treatment

### 2.1. Endoscopic Intralesional Injection of Steroid Therapy

Steroids can inhibit inflammation and fibrosis. The endoscopic intralesional injection of triamcinolone acetonide (TA) has been used for the treatment of benign and malignant esophageal strictures [15, 16]. Hashimoto et al. found that the endoscopic injection of TA after ESD in 41 patients with mucosal defects of three-quarters of the esophageal circumference was safe and effective to prevent esophageal stricture [17]. Twenty-one patients in the treatment group had an endoscopic injection of TA on the 3rd, 7th, and 10th days after ESD with a dose of 16–62 mg of TA for each treatment. The incidence of stricture in the treatment group (19.0%) was apparently lower than that in the control group without TA injection (75.0%), and the number of extra EBD procedures to treat the stricture decreased. Hanaoka et al. also reported a prospective study in 30 patients who had a single injection of 100 mg TA immediately after ESD [18]. The stricture rate was 10%, which is lower than that in the historical control group of 29 patients without TA injection (66%). However, Takahashi et al. showed that in patients with a circumferential mucosal defect of more than three-quarters of the circumference of the esophagus, it was difficult to prevent refractory stricture, despite the patients receiving TA injection after ESD [19]. The stricture rate was not significantly different, from 87.5% in the control group to 62.5% in the study group. Additionally, the perforation rate during dilatation procedures was 1.0% in the study group but 0.5% in the control group. Hanaoka et al. confirmed that a tumor extent greater than 75% of the esophageal circumference was an independent risk factor for complex stricture [20]. Nagami et al. reported in a retrospective matched case-control study of 602 patients that a single injection of TA after ESD effectively reduced the esophageal stricture rate and the number of EBD sessions [21]. However, the efficacy reduced in patients with entire circumferential mucosal defects. Steroid treatment concerns include the possibility of periesophageal abscess after steroid treatment and the increased risk of delayed perforation in the extra EBD procedures [22, 23].

### 2.2. Steroid Gel Therapy

An improper endoscopic intralesional injection carries the risk of bleeding and myofiber atrophy because TA must be injected in the submucosa. Therefore, Mori et al. changed the application method of TA by a prospective study [24]. Twenty patients received a 17.5 mL TA gel treatment applied to the ulcer floor and 5 minutes of balloon dilatation to permeate the steroid on the 5th, 8th, 12th, and 15th days after ESD. The control group accepted TA injection and balloon dilatation after ESD. The stricture rate had no significant difference between the two groups. The TA gel application is safe and effective to prevent esophageal stricture after ESD, but visibly, it has too many operation procedures.

### 2.3. Oral Administration of Steroid Therapy

The oral administration of steroids has also been widely researched to prevent esophageal stricture due to their anti-inflammatory effects. Yamaguchi et al. reported the effectiveness for stricture prevention in the study of 41 patients who underwent more than three-quarters of circumference circular ESD [25]. Twenty-one patients in the study group received oral prednisolone starting on the third day after ESD. The dose of prednisolone was 30 mg/d for the first two weeks, 25 mg/d for the next two weeks, and then was gradually decreased to 5 mg/d each week over the next four weeks until termination, eight weeks after ESD. The stricture rate in the study group (5.3%) was lower than that in the control group (31.8%). The control group performed preemptive EBD twice a week for 8 weeks after ESD. Isomoto et al. reported similar conclusions in patients with complete, circular ESD [26]. Sato et al. evaluated a retrospective cohort study and found that early administration of oral prednisolone combined with EBD is an effective method to prevent esophageal stricture after ESD and early steroid administration is better than late administration or no steroid therapy [27]. However, it is possible for some systemic problems to appear after long-time oral steroid therapy, such as peptic ulcers, immune suppression, metabolic disturbances, and psychiatric symptoms. Kataoka et al. shortened the period of steroid use in their research [28]. Seventeen patients in the study group underwent prednisolone treatment from the third day after ESD, at a dose of 30 mg/d during the first one week. Then, the dose was gradually decreased to 10 mg/d every week for the next two weeks until termination, three weeks after ESD. The patients in the two groups showed no significant differences, but the incidence of esophageal stricture was lower in the study group than in the ESD-alone group (17.6% versus 68.7%, resp.). Recent studies showed that oral administration of steroids has little adverse events or serious complications. However, there is a study that reported a nocardiosis infection in an elderly patient who received oral steroid treatment after ESD [29].

### 2.4. Antifibrotic Drug Therapy

Some antifibrotic drugs have been studied in the prevention of esophageal stricture. Antifibrotic drugs inhibit the proliferation of fibrous scars. 5-Fluorouracil (5-FU) is an antineoplastic drug. 5-FU combined with TA was researched in regard to the reduction of strictures that occur after subepiglottic surgery [30]. Mizutani et al. reported that 5-FU can be used as an antiscarring agent [31]. In their research, 5-FU was combined with liposome and mixed with atelocollagen, for sustained release. It was effective in preventing esophageal strictures after ESD in a canine model, by reducing submucosal fibrosis.

Tranilast is an antiallergic drug that can inhibit the release of transforming growth factor-beta (TGF-*β*1), prostaglandin-E_2_ (PGE2), and interleukin-1 (IL-1), which reduces collagen synthesis and fibrosis [32]. Tranilast was used to treat keloids and hypertrophic scars in an animal study [33]. Uno et al. reported a pilot study that demonstrated the availability and safety of oral tranilast with EBD to prevent esophageal strictures after ESD [34].

N-acetylcysteine is an antioxidant that inhibits TGF-*β*1. However, the use of N-acetylcysteine failed to reduce the formation of esophageal fibrogenesis after circumferential ESD in a pig model [35].

Botulinum toxin type A (BXT-A) is a neurotoxin that inhibits the deposition of collagen fibers and improves hypertrophic scars [36]. Wen et al. proved that BTX-A can reduce esophageal strictures in patients who underwent more than two-thirds of circumference circular ESD [37]. The endoscopic intralesional injection of BTX-A after ESD can reduce stricture rates to 6.1%, compared with 32.4% in the control group.

## 3. Esophageal Stent Treatment

### 3.1. Esophageal Self-Expandable Stents

Metallic esophageal stents function to expand the esophagus. Metallic esophageal stents were initially researched for the interventional treatment of esophageal fistulas and esophageal strictures caused by malignant esophageal neoplasms [38, 39]. The application of metallic esophageal stents in benign esophageal strictures is controversial, due to common adverse reactions such as bleeding, esophageal perforation, stent migration, or stricture recurrence [39, 40]. Comparatively speaking, temporary self-expandable metallic stents are more suitable to be used in the treatment of benign esophageal strictures [41, 42]. A positive aspect of these stents is that they can be removed easily; however, a negative aspect is the high recurrence rate after stent removal [40, 43]. Several studies reported that elderly patients with refractory cicatricial strictures after ESD had temporary self-expandable metal stents placed, which resulted in successful esophageal treatment without complications (i.e., fever, chest pain, or stricture recurrence) [44, 45]. The efficacy of circumferential esophageal stents for the prevention of stricture formation after ESD has been reported by Wen et al. [46]. In their randomized controlled trial, 22 patients with a circumferential mucosal defect of more than three-quarters of the esophageal circumference were included. The study group had esophageal stents for 8 weeks and had a significantly lower stricture rate (18.2%) than the no-stent group (72.7%). The complication of stent migration into the stomach still occurred, which markedly reduced the expansion effect of the stents and required a repeat endoscopy operation to reposition the stent. The long-term stricture-preventing effects after the removal of the stents were unknown.

### 3.2. Biodegradable Stents

Some researchers attempted to use biodegradable stents to treat benign esophageal strictures [47]. Compared to metallic or plastic stents, biodegradable stents have the advantages that they do not need to be removed and have sufficient radial force to expand esophagus [44, 48]. Poly-L-lactic acid (PLLA) is one kind of biodegradable stent that was reported to prevent restricture in two patients after near-circumference ESD [49]. Lua et al. reported on biodegradable stents made of carboxymethyl cellulose (CMC) [50]. Seven patients with mucosal defects of three-quarters of the esophageal circumference had endoscopic placement of CMC stents after ESD. This research had no control group. The stricture rates were approximately 57%. Compared to the other research, the preventing efficacy of CMC esophageal stents appears to be limited.

Polyglycolic acid (PGA) sheets have been used in implantation surgeries to reinforce sutures [51]. In recent years, PGA sheets have been used to repair mucosal defects, prevent scar contracture, and alleviate postoperative pain [52–54]. The combination of PGA sheets and fibrin glue as an endoscopic tissue shielding method has been used in the colon and duodenum, with few postoperative adverse events [50, 55]. Iizuka et al. demonstrated the potential of PGA and fibrin glue for the prevention of stricture after ESD [56]. Fifteen patients with defects greater than half of the circumference after ESD were included in the study, and 6 weeks later, the esophageal stricture rate was 7.7%. The small PGA sheets (15 × 7 mm) were placed on an artificial ulcer without overlapping, and fibrin glue was sprayed to affix the PGA. Limitations of this method include that the small sheets take a long time to place and are easy to drop. Thus, Ono et al. reported a novel technique called “the clip and pull method”, using a whole PGA sheet to shield an artificial ulcer [57]. This method was used in eight patients with a circumferential mucosal defect of more than three-quarters after ESD [58]. The stricture rate was 37.5%, and the number of EBD sessions was 0.8 ± 1.2. Additionally, Kataoka et al. reported a case of an elderly patient that did not suffer dysphagia after circumferential ESD, by the treatment of the steroid injection, shielding the ulcer with PGA and fibrin glue [59]. The combination therapy of intralesional steroid injection and PGA sheets also showed positive effects on ten patients after near-circumference ESD [60]. Although further research is needed to confirm these findings, the results from these studies increase the interest in the combination therapy of PGA sheets and other treatments.

## 4. Tissue Engineering Approaches

### 4.1. Extracellular Matrix Scaffold Therapy

Extracellular matrix (ECM) scaffolds can support the growth of epithelial cells, are compatible with perivascular stem cells, and promote wound recovery and esophageal structure remodeling [61–63]. The ECM scaffolds derived from the small intestinal submucosa or urinary bladder submucosa were reported to achieve reconstruction of the esophagus in a dog model [64]. Then, Badylak et al. reported that the ECM scaffolds can minimize stricture and promote esophageal remodeling in five male patients after endoscopic inner-layer circumferential resection [65]. The actual esophageal remodeling mechanisms in patients were unclear, but the study showed that cryptic peptides formed in scaffold degradation maybe the potential factor. Nieponice et al. researched a dog model to evaluate the potential of urinary bladder ECM tubular scaffolds for the prevention of esophageal stricture [66]. Five dogs had endoscopic ECM scaffold placement after circumferential esophageal EMR, while another five dogs only had circumferential esophageal EMR. As a result, histological assessment of the ECM treatment group showed a continuous, intact, regenerate esophageal mucosa with no inflammation or necrosis, while histology of the control group showed an immature epithelial layer with inflammation and severe scarring. A surgical adhesive was used in this study to prevent scaffold migration, but the influence of this adhesive on esophageal mucosal remodeling was unclear. Additionally, the efficacy of the ECM scaffold was controversial. Schomisch et al. reported an unsuccessful study on the prevention of stricture formation using three ECM scaffolds: the small-intestine submucosa, acellular dermal matrix, and urinary bladder matrix [67]. The major influencing factors of the study included the preparation technique of the scaffold and the use of a self-expanding stent rather than the use of surgical adhesive to attach the scaffold. Future biological ECM research may focus on novel materials and proper techniques to promote the recovery of the esophagus.

### 4.2. Cell-Based Therapy

Cell transplantation is applied to esophageal mucosal defects, inducing early reepithelization and promoting scarless wound healing. The efficacy of implantation of autologous keratinocytes or adipose stromal cells was proven in several animal model studies [68–70]. However, the long-term efficacy of the direct injection of the cells was unknown. The cells that migrated after injections were difficult to trace for prolonged periods. Additionally, the real mechanism of the reepithelization is still uncertain.

A new approach using endoscopic transplantation of cultured autologous cell sheets overcame the limitations of cell migration and low viability rates of transplanted cells. The cell sheets are fabricated on a temperature-responsive culture surface and can be easily harvested. The sheets can be transplanted to the target sites without the use of sutures or adhesives [71]. The transplantation of cell sheets after ESD can contribute to early epithelium regeneration and mild fibrosis. However, much of this area of research was performed in animal models. Kanai et al. proved that the epidermal cell sheets can reduce the symptom of dysphagia in patients who underwent circumferential ESD [72]. Perrod et al. reported that adipose tissue-derived stromal cell sheets can reduce the stricture rate after ESD [73, 74]. In animal and clinical research, cultured autologous oral epithelial cell sheets can suitably cover ulcer areas and effectively reduce the degree of stricture [75–78]. Ohki et al. demonstrated that transplantation of autologous oral epithelial cell sheets can safely and effectively prevent esophageal stricturing and promote epithelial healing after ESD without the need for additional treatments for complications [76, 78]. The growth factors, cytokines, and the source of regenerated epithelia may be related to the early reconstruction of the esophageal surface [75]. In further studies, more clinical evaluation and long-term follow-up should be performed to ensure the safety and reproducibility of the cell sheet technique. The high cost of fabrication and rapid adhesion warrants further research of methods to facilitate the transplantation of the cell sheets. In addition, some methods demonstrate the potential of cell-based therapy for the prevention of postoperative strictures. Mizushima et al. showed that the application of conditioned medium obtained from mesenchymal stem cells, combined with the injection of steroids, can significantly decrease inflammation and fibrosis of the animal esophagus after ESD [79].

## 5. Conclusion

In brief, we reviewed recent publications on the prevention of esophageal stricture after ESD, all of which inevitably have their own limitations and cannot be widely accepted in clinical application (Table 1). The steroid therapies have been effective in many clinical trials but cannot completely prevent stricture in some high-risk patients. The reduced efficacy of these therapies to prevent stricture after near-circumference or whole-circumference ESD is an ongoing problem. Thus, a sufficient evaluation before endoscopic surgery and a prolonged assessment after preventive therapy are essential to current comprehensive treatment. The combination of different therapies should be evaluated in future studies.

In recent years, many innovative therapies have shown appreciable feasibility, but they still require controlled clinical research to confirm effectiveness. Some single case reports lack consensus, needing more evidence to sufficiently confirm safety. After the improvement of current therapies, ESD can be more widely utilized as a minimally invasive treatment with minimal complications.

## Figures and Tables

**Table 1:** Treatments for the prevention of esophageal stricture after endoscopic submucosal dissection.

Group		Mechanisms	Advantages	Disadvantages and limitations
Pharmacological treatment	Steroid	Anti-inflammatory, antifibrotic formation, antiscar formation	Effective in many small comparative clinical studies	Hard to prevent stricture in patients with circumferential esophageal mucosal defects, systematic side effects (peptic ulcers, immune suppression metabolic disturbances, and psychiatric symptoms), and delayed wound healing
	Antifibrotic drug	Inhibit the proliferation of fibrous scars	Antifibrotic effect	No randomized controlled trials or systematic reviews with sufficient evidence
Esophageal stent treatment	Esophageal self-expandable stents	Expand the esophagus	Persistently expand the esophagus, easily to be removed at any time	Adverse reactions (bleeding, chest pain, esophageal perforation, and stent migration), high recurrence after stent removal, and long-term effects were unknown
	Biodegradable stents	Expand the esophagus	Expand the esophagus, no need to remove	No randomized controlled trials or systematic reviews with sufficient evidence
Tissue engineering approaches	Extracellular matrix scaffold	Support the growth of epithelial cells, promote esophageal structure remodeling	Support tissue, enhance mucosal healing and structure remodeling	Potential safety problem, no randomized controlled trials, or systematic reviews with sufficient evidence
	Cell-based therapy	Promote reepithelialization and scarless wound healing	Reepithelialization, enhancement of mucosal healing and structure remodeling, great potential for development	Complicated technique, high cost, large-sample controlled trial, and long-term follow-up research are needed
